# DNA vaccination for prostate cancer: key concepts and considerations

**DOI:** 10.1186/s12645-015-0010-5

**Published:** 2015-07-02

**Authors:** Grace Cole, Joanne McCaffrey, Ahlam A. Ali, Helen O. McCarthy

**Affiliations:** School of Pharmacy, Queen’s University Belfast, 97 Lisburn Road, Belfast, BT9 7BL Northern Ireland UK

**Keywords:** Prostate cancer, DNA vaccine, Prophylactic, Therapeutic, Tumour associated antigens

## Abstract

While locally confined prostate cancer is associated with a low five year mortality rate, advanced or metastatic disease remains a major challenge for healthcare professionals to treat and is usually terminal. As such, there is a need for the development of new, efficacious therapies for prostate cancer. Immunotherapy represents a promising approach where the host’s immune system is harnessed to mount an anti-tumour effect, and the licensing of the first prostate cancer specific immunotherapy in 2010 has opened the door for other immunotherapies to gain regulatory approval. Among these strategies DNA vaccines are an attractive option in terms of their ability to elicit a highly specific, potent and wide-sweeping immune response. Several DNA vaccines have been tested for prostate cancer and while they have demonstrated a good safety profile they have faced problems with low efficacy and immunogenicity compared to other immunotherapeutic approaches. This review focuses on the positive aspects of DNA vaccines for prostate cancer that have been assessed in preclinical and clinical trials thus far and examines the key considerations that must be employed to improve the efficacy and immunogenicity of these vaccines.

## Introduction

Prostate cancer represents a major challenge to healthcare and accounts for 25 % of all new diagnoses in males in the UK annually [1]. Localised prostate cancer may be treated with prostatectomy or radiotherapy, which aims to remove or reduce the tumour load and is associated with favourable overall survival [2, 3]. However, typically anywhere from 20–30 % of patients experience a recurrence or present with locally advanced or metastatic disease [4]. The first line treatment for these patients is androgen deprivation therapy (ADT) which is associated with unpleasant side-effects such as urinary and erectile dysfunction [1, 3], and after an initial response, the majority of cases eventually progress to castration resistant prostate cancer (CRPC). Docetaxel is the gold standard treatment for CRPC but is not curative and is associated with only a moderate (2.4 months) survival advantage [5, 6]. As such, there is a clinical need for newer, highly effective treatment options for patients with CRPC.

Immunotherapy is a strategy for cancer treatment that has received increasing attention over the last few decades. The goal of immunotherapy is to harness the immune system to mount a response against tumour associated antigens (TAAs), normal proteins expressed by or upregulated in cancer cells [7]. In order to be successful the vaccine must be capable of generating a tumour specific T cell response to weakly immunogenic “self-antigens” [7, 8]. The vaccine must also overcome the mechanisms of immune evasion employed by cancer cells, such as, the immunosuppressive microenvironment, downregulation of major histocompatibility complex (MHC) antigen presentation, upregulation of regulatory T cells and co-inhibitory signalling pathways [8, 9].

Prostate cancer is an ideal candidate for immunotherapy for a number of reasons. For example, the slow growing nature of cancer within the prostate [10] allows sufficient time for the immune system to mount an anti-tumour response following a prime/boost or multiple immunisation strategy. In addition, prostate cancer expresses numerous TAAs which include the Prostate Specific Antigen (PSA) [11, 12], Prostatic Acid Phosphatase (PAP) [13], Prostate Specific Membrane Antigen (PSMA) [12, 14], Prostate Stem Cell Antigen (PSCA) [15] and Six Transmembrane Epithelial Antigen of the Prostate (STEAP) [16]. All of these TAA’s provide multiple potential immunological targets [17] and indeed the ideal combination of antigens has yet to be elucidated. Furthermore, the presence of PSA in patient serum enables the malignancy to be detected early and in some cases even before tumours are radiologically detectable [9]. This in turn facilitates earlier treatment [18]. Circulating T cells that react with prostate TAAs have previously been detected, which suggests that self-tolerance towards these antigens can be overcome [18]. The prostate is considered to be a non-essential organ and therefore immunological treatments utilising prostate TAAs will not cause acute off-target toxicity [9, 18]. Finally and perhaps most importantly, the first prostate cancer specific immunotherapy, Sipuleucel-T (Provenge®, Dendreon Corporation, Seattle, WA), has recently been licensed by the US Food and Drug Administration (FDA) in 2010 for asymptomatic or minimally symptomatic CRPC [19]. Sipuleucel-T consists of autologous peripheral blood mononuclear cells with antigen presenting dendritic cells that have been activated *ex vivo* with a recombinant fusion protein (PA2024) consisting of PAP linked to granulocyte-macrophage colony stimulating factor (GM-CSF) [19]. In a phase III trial, CPRC patients receiving Sipuleucel-T had a 22 % reduction in mortality [20]. The success of the therapeutic Sipuleucel–T has paved the way for other immunotherapeutic prostate cancer vaccines to be granted regulatory approval and enter the market.

Other immunotherapeutic cancer vaccine approaches which have been clinically investigated for prostate cancer include the administration of whole tumour cells [21], dendritic cells (DCs) loaded with peptides or tumour cell lysate [22], peptide vaccines [23] and the administration of antibodies [24]. This review examines the progress of DNA vaccines specifically for prostate cancer and focuses on the key considerations required for successful development. Only the most recent studies are included in this review to bring the reader up to date with the field. Clinical trials that utilise DNA vaccines in prostate cancer therapeutically are summarised in Table 1, while DNA vaccines administered prophylactically in preclinical models prior to tumour challenge are summarised in Table 2. In addition, ongoing Phase II or III clinical trials utilising DNA vaccines in prostate cancer are detailed in Table 3.

**Table 1:** Summary of therapeutic clinical trials utilising DNA vaccines for prostate cancer

Vaccine/ targeted antigen	Phase trial/ number of patients	Delivery route	Immunological response/ clinical outcome/PSA DT	Ref
PAP: pTVG-HP (100 μg) with rhGM-CSF (200 μg)	Phase II (NCT00849121) *N* = 17	i.d	No. with tripling of T-Cell specific antibodies- Group 1: 3/8 Group 2: 6/8	[99]
			No. with doubling of PSA DT- Group 1: 3/8 Group 2: 4/9	
PAP: pTVG-HP (100 μg, 500 μg or 1500 μg) with GM-CSF (200 μg)	Phase I/IIa (NCT00582140) *N* = 22	i.d.	No. with PAP-specific IFNγ-secreting CD8+ T-cells- 3/22	[31]
			No. with tripling of CD4+ and/or	
			CD8+ T-cell proliferation – 9/22	
			No. with doubling of PSA DT- 7/22	
PSA: PROSTVAC with GM-CSF (100 μg)	Phase II (NCT00078585) *N* = 125	s.c.	Overall survival- PROSTVAC group: 25/82 Control: 7/40	[75]
			Median survival- PROSTVAC group: 25.1 months Control: 16.6 months	
PSA: Ad/PSA (10^6^, 10^7^, 10^8^pfu)	Phase I (IND #9706) *N* = 32	s.c.	No. with anti-PSA T cell responses- 15/28	[74]
			No. with increased PSA-DT- 13/28	
PSA: pVAXrcPSAv531 (rhPSA) (50–1600 μg)	Phase I (NCT00859729) *N* = 15	i.d. with EP (DERMAVAX)	No. with prolongation of PSA-DT by at least 50 % during study- 4/15	[47]
PSMA: DOM-PSMA_27_ (800–3200 μg)	Phase I/II *N* = 30	i.m. with or without EP	No. with detectable anti-PSMA_27_ CD8+ T cells response- 16/30	[48]
			No. with doubling of PSA-DT- 14/30

**Table 2:** Summary of preclinical prophylactic prostate cancer tumour challenge studies utilising DNA vaccines

Vaccine/ targeted antigen	Model	Delivery route	Clinical outcome	Ref
PSCA/STEAP: pCI-neo-mPSCA and/or pCI-neo-mSTEAP1 (100 μg) prime plus MVA-mPSCA and/or MVA-mSTEAP1 (1X10^7^ pfu) boost	C57 BL/6	i.m. prime	Significant reduction in tumour volume	[17]
	TRAMP C-1	i.p. boost	Significant delay in time to form tumours	
hPSA: phPSA (50 μg) with or without CpG	C57 BL/6	i.m. with EP	Significant delay in appearance of tumours	[46]
	TRAMP C-1/hPSA		Significantly prolonged survival	
PSMA/PSCA/STEAP: rAd/PSMA, rAD/PSCA, rAd/STEAP prime (1X10^8^ PFU); TRAMP C-1 pulsed DCs (2X10^6^ cells) boost	C57 BL/6	i.v. prime	Tumour Growth Significantly delayed	[112]
	TRAMP C-1	s.c. boost		
STEAP: mSTEAP DN A (2 μg) prime with: mSTEAP DN A (2 μg) or mSTEAP-VRP (10^6^ IU) boost; or mSTEAP-VRP (10^6^ IU) prime and boost	C57 BL/6	i.d. (gene gun)	Significantly prolonged survival	[56]
	TRAMP C-2	s.c.	Significantly delayed tumour growth

**Table 3:** Summary of ongoing or unpublished clinical trials utilising DNA vaccines for prostate cancer

Vaccine/ targeted antigen	Phase trial/ estimated enrolment	Delivery route	Primary objectives	Ref
PAP: Sipuleucel-T with or without pTVG-HP (100 μg)/ rhGM-CSF (200 μg)	Phase II (NCT01706458) *N* = 30	i.d.	PAP-specific Immunological response	[113]
PAP: rhGM-CSF (200 μg) with or without pTVG-HP (100 μg)	Phase II (NCT01341652) *N* = 56	i.d.	Metastasis-free survival	[114]
PSA: PROSTVAC with or without GM-CSF (100 μg)	Phase III (NCT01322490) *N* = 1200	s.c.	Overall Survival	[77]
PSA: Flutamide with or without PROSTVAC	Phase II (NCT00450463) *N* = 53	s.c.	Time to Treatment Failure	[115]
PSA: Adenovirus/PSA (1X10^8^ pfu in gelfoam)	Phase II (NCT00583024) *N* = 66	s.c.	PSA-DT Response	[76]
PSA: Adenovirus/PSA (1X10^8^ pfu in gelfoam) with or without ADT	Phase II (NCT00583752) *N* = 70	s.c.	PSA-DT Response	[116]

### DNA vaccines

**Fig. 1:**
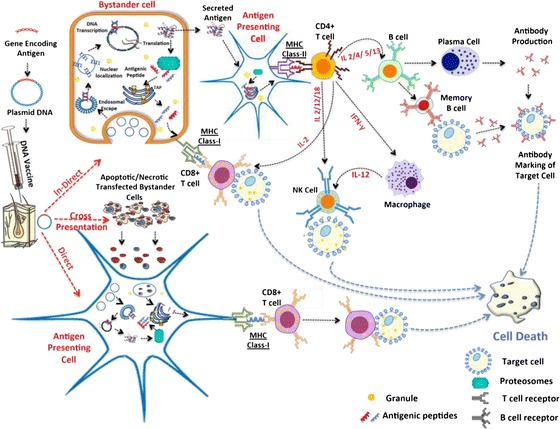
Schematic representation of immune responses elicited following DNA vaccination. DNA may be taken up by bystander cells (e.g., muscle cells, keratinocytes) or APCs at the site of immunisation resulting in production of host-synthesised antigens capable of eliciting immune responses via both MHC‐I and MHC‐II pathways. APCs have a central role in the induction of immunity following vaccination, either by direct transfection of the APCs or cross-presentation through bystander cell associated exogenous antigens resulting in presentation of antigen on MHC class-I molecules, eliciting CD8+ T cell expansion. Additionally, exogenous antigens, secreted from bystander cells, captured and processed by APCs are presented via MHC class‐II molecules resulting in CD4+ T cell expansion resulting in a cascade of cellular responses and B cell activation and antibody production

One of the key goals in a cancer vaccine is to induce a cell-mediated immune response, primarily through the activation of TAA-specific cytotoxic T lymphocytes (CTLs). Therapeutic responses arise from activation of these antigen specific CTLs which cause destruction of TAA expressing cells. This could potentially eradicate disseminated deposits of prostate cancer for which current treatment options are limited following the onset of castrate resistance. It has been well documented that DNA vaccination is a highly potent strategy for inducing both prophylactic and therapeutic responses [25]. However, in order for the desired antigen to be expressed the plasmid DNA needs to be delivered to the nucleus of the cell. There is also a need to ensure that the DNA is delivered to antigen-presenting cells (APCs), so that the antigen expressed in the cytoplasm can be presented via the MHC class I complex [26]. Presentation via the MHC class I complex will result in a much more potent cell-mediated therapeutic immune response. Additionally, APCs are capable of internalising and processing exogenous antigens from neighbouring apoptotic cells that have been transfected. Such antigens are again presented via the MHC class I pathway and this process is known as cross-presentation [26]. DNA that is delivered to non-APCs will give rise to exogenous antigens that activate the more common MHC class II pathway which is characterised by a humoral response and a subsequent prophylactic action (Fig. 1).

DNA vaccines confer many advantages over conventional treatments: (1) DNA vaccines are capable of eliciting host humoral and cellular immunity, leading to a potent, wide-sweeping immune response to TAAs [25]; (2) Genes encoding the full length of the TAA can be introduced, ensuring that the correct post-translational modifications occur in the cell, thus presenting multiple potential antigenic epitopes to the immune system [27, 28]; (3) The TAAs confer high specificity which renders DNA vaccines safe compared to conventional treatments [29] and safety has been demonstrated in animals and in several clinical trials [30, 31]; (4) DNA is relatively easy to produce and purify and is highly reproducible, therefore DNA vaccines should be cost-effective for large scale manufacture [27, 29]; (5) Lastly, in contrast to conventional live attenuated vaccines there is no reversion risk to pathogenicity *in vivo*.

The worldwide DNA vaccine market is projected to increase to $2.7 billion by 2019, yet there are only four commercially available DNA vaccines licensed for use and these are in animals. Licensed vaccines include the West Nile-Innovator® DNA (Pfizer), Apex®-IHN (Novartis Aqua Health), ONCEPT™ (Vical) and LifeTide® SW 5 (VGX Animal Health). Of these DNA vaccines only ONCEPT™ has been licensed (2007) for use in cancer, specifically for the treatment of malignant melanoma in dogs [32]. Despite the promise shown by DNA vaccines in preclinical models, success has proven difficult to reproduce in larger animals and clinical trials [33]. This lack of efficacy is thought to be due to low immunogenicity and cellular uptake of DNA. Nevertheless, if the barriers preventing the translation of this therapy to humans can be overcome, the impact of DNA vaccination on the treatment of cancer could be revolutionary.

**Fig. 2:**
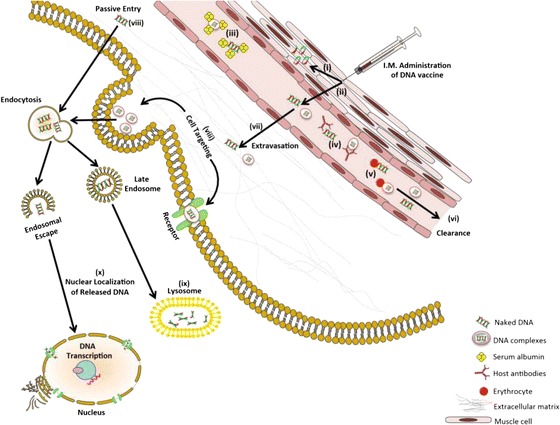
Schematic representation of extracellular and intracellular barriers to DNA delivery. DNA and DNA complexes delivered *in vivo* must overcome a number of barriers to achieve successful gene expression in the cell nucleus: (i) Endo and exonuclease degradation of DNA; (ii) Migration of DNA from the target tissue into systemic circulation; (iii) Binding and aggregation of DNA via serum protein complexation; (iv) Immune activation to delivered DNA; (v) Interaction and binding with erythrocytes; (vi) Clearance of DNA via spleen, renal and hepatic systems; (vii) Migration of DNA through extracellular matrix in target organ; (viii) Cellular uptake, mediated via endocytosis or passive entry; (ix) enzymatic degradation of DNA in lysosome; (x) Nuclear localization of DNA for protein expression

In order to be efficacious, DNA must reach the cell nucleus in quantities sufficient to produce enough antigen to overcome self-tolerance. After introduction to the host there are a number of biological barriers to nuclear delivery that contribute to a low clinical success rate. At the cell surface DNA must undergo internalisation across the cell membrane, which frequently results in endosomal entrapment. In the endosome, DNA is vulnerable to degradation by intracellular nucleases and must escape into the cytoplasm. From the cytoplasm, the DNA must be actively transported into the cell nucleus where the cell can begin to transcribe and translate the DNA to produce the antigen of interest [34–36]. Vectors can be utilised to improve DNA delivery. Such vectors can be employed to condense and protect DNA from clearance and degradation in addition to overcoming the extra and intracellular barriers (Fig. 2).

### Strategies to improve DNA vaccine efficacy

A number of factors contribute to the overall transfection rate and therefore efficacy of each DNA vaccine. With a plethora of delivery platforms and strategies designed to improve the potency of DNA vaccines, it is difficult to elucidate the optimum delivery strategy for the “best” TAA. Few studies include a direct comparison between the efficacy of a delivery system against the current gold standard, with most studies examining a new delivery vehicle against control groups receiving “naked” DNA or no treatment. This makes it particularly difficult to evaluate the true potential of any new delivery strategies. This is further complicated by discrepancies in experimental design and evaluation, which render it almost impossible to directly compare the variety of approaches employed.

Injection of “naked” DNA is the simplest delivery strategy and has been shown to induce humoral and cellular immune responses when administered to mouse models [37]. However, this strategy offers little protection to DNA and transfection rates are significantly reduced when upscaled to human studies [30]. Several delivery approaches are undergoing investigation to improve DNA vaccine efficacy. Delivery platforms can be broadly classified as physical or non-physical (vector-based) methods, which can be further subcategorised to either viral or non-viral. In addition to the DNA delivery platform, consideration must be given to the immunisation protocol, the co-administration of adjuvants, which may be used to modify the cellular environment, and to the origin and combination of DNA delivered which may play a central role in the induction of a potent immune response. This review shall introduce and provide a brief discussion of the most recent developments in each field, but shall focus on the strategies that are most applicable to prostate cancer.

#### Physical delivery methods

Physical delivery methods act to overcome the extracellular barriers to gene delivery and/or to temporarily disrupt the membrane of target cells, allowing DNA entry. Tattooing [38], micro-injection [39], gene gun [40], ultrasound [41] and electroporation (EP) [42] have been described as physical methods for gene delivery. Of these, EP, gene gun and ultrasound have been used for gene delivery in prostate cancer models.

#### Electroporation

EP is a technique whereby DNA is delivered intradermally (i.d.) or intramuscularly (i.m.) to the target site and a short electrical pulse or series of electrical pulses are applied locally to the area. This results in a transient destabilisation of cell membranes in the target tissue [35]. EP has been well documented as a potent means of enhancing transgene delivery, with antigen expression reported to increase up to 1000 fold [28, 43, 42]. Antigen specific responses have also been detected 25 weeks post immunisation [44]. A favourable safety profile also makes EP an attractive option for *in vivo* immunisation.

EP has been used to enhance DNA vaccine immunogenicity in several prostate cancer preclinical models. For example, Roos *et al.* demonstrated a significant increase in PSA specific CD8+ T cells circulating in peripheral blood following i.d. injection of only 10 μg pVax-PSA accompanied by EP compared to those receiving no EP in C57 BL/6 mice following 2 immunisations [45]. EP has also demonstrated efficacy and safety in a number of clinical trials [12, 46, 47]. For example, in a phase I/II dose escalation trial, patients with biochemically recurrent prostate cancer were immunised i.m. with pDOM-PSMA, a DNA fusion vaccine encoding a PSMA epitope, PSMA_27_, and DOM, a domain of fragment C a tetanus toxin, without (Arm I) or with (Arm II) EP [48]. Patients received a total of five immunisations at weeks 0, 4, 8, 24 and 48, with follow-up recorded up to week 72. At week 24, 11 of the 15 patients from Arm I switched to Arm II due to a significantly higher anti-DOM antibody response in patients receiving EP [48]. Vaccination with or without EP was associated with significant increases in detectable DOM-specific CD4+ and PSMA_27_-specific CD8+ T cells compared to baseline, with a significant trend towards higher responses in those treated with EP up to week 24 [48]. Treatment was associated with a significant increase in PSA doubling time (PSA-DT), an indication of disease progression, from 11.98 months pre-treatment to 16.82 months at 72 week follow-up, independent of whether the patient had received EP or not. While the authors found in this case that vaccination and EP were well tolerated by patients, other reports have found that EP is associated with pain, inflammation and bleeding, especially when given i.m. which could decrease clinical acceptability [49, 50]. It is likely that the delivery site (whether i.d. or i.m.) is key to the generation of the immune response. For example, the high population of resident APCs in the skin have increased the number of EP plus i.d. studies [42]. Eriksson *et al.* delivered pVAX plasmid encoding rhesus macaque PSA (rhPSA), pVAX/rhPSA, to patients with biochemical evidence of prostate cancer relapse i.d. followed by EP with the DermaVax device (BTX/Harvard Appartus) and monitored patients for evidence of decrease in PSA-DT or generation of PSA-specific T cells [47]. However, no significant changes in PSA kinetics were observed in any patients and increased PSA-specific T cell reactivity was only observed in patients in the highest dose cohorts (4 of 15) [47]. The authors speculated that i.m. delivery elicits a more potent immunological response. However, as this is more invasive it may be desirable to increase the potency of i.d. vaccination with higher vaccine doses or with the use of adjuvants [47].

To conclude, EP is a well established means of enhancing transgene expression and acts as an immune adjuvant [42] due to the inflammation and recruitment of DCs following application, largely due to local tissue damage. Damage is directly related to the intensity of EP and higher intensities are associated with higher transfection efficacies [42]. As such, there is a trade-off between increased efficacy and discomfort to the patient, with the latter being the rate-limiting factor [42]. Despite these concerns EP has been used safely in a number of key prostate cancer clinical trials [47, 48], providing an encouraging platform for DNA vaccine delivery. The availability of commercially produced EP devices also provides a convenient, reproducible means for researchers to administer their vaccines in preclinical and clinical trials.

#### The gene gun

A second physical delivery strategy is the use of the “gene gun”, whereby naked DNA is adhered to the surface of gold particles, which are accelerated under high pressure by a ballistic device into the target tissue. Such high pressure is necessary to ensure penetration of cell membranes which is vital for intracellular DNA delivery [40]. The gene gun has demonstrated superior gene expression compared to injection of naked DNA [51], and is capable of enhancing specific humoral and cellular immunity [51]. In preclinical trials the gene gun has also demonstrated comparable efficacy to EP in inducing a potent cellular immune response following i.m. injection [52]. As such, the gene gun has been used to induce antigen specific responses in several clinical trials [53, 54], most notably to protect humans from influenza challenge [54]. However, its use as a delivery agent in clinical trials for cancer treatment has been less successful, perhaps due to limited gene carrying capacity (~2 μg per dose), which often necessitates multiple immunisations at different sites in the body, reducing patient compliance [52–54]. Despite this dose limitation the gene gun is a simple and flexible device for *in vivo* gene delivery and has been used for the delivery of prostate TAAs in several preclinical studies. Gregor *et al.* [55] and Garcia-Hernandez *et al.* [56] have used the gene gun to deliver prostate TAAs in murine preclinical studies and these are discussed subsequently.

To conclude, the gene gun is a flexible, fast and highly reproducible option for *in vivo* gene delivery due to the availability of a commercial device (Helios Gene Gun, Bio-Rad). The gene gun is however limited by the DNA carrying capacity, as well the degree of tissue penetration, with penetration depths limited to 0.1 mm [40]. Therefore, moving to larger animal models and humans may prove impractical.

#### Ultrasound

Although EP and gene gun have good efficacy *in vivo* there are newer, less invasive physical delivery systems being developed. One such delivery enhancement strategy is ultrasound. DNA is injected into the target tissue and ultrasonic waves are applied externally, continually or in pulses, causing a transient, reversible increase in cell membrane permeability, thus facilitating cellular entry of the DNA [41, 57]. Transfection efficacy varies according to a number of factors including frequency, intensity and duration of application. However, optimal conditions have not yet been established and the risk of cellular damage to the host tissue has not been fully elucidated [41]. Although ultrasound has been shown to increase gene expression 10–15 fold *in vivo* compared to “naked” DNA [39], levels of gene expression are still considerably lower than that which can be achieved using either EP or gene gun approaches [39]. Nevertheless, Yoshida *et al.* [58] utilised ultrasound to enhance delivery of mannose-modified bubble lipoplexes containing ubiquitylated melanoma-related antigen (pUb-M) to APCs. In combination with doxorubicin, this produced a robust CTL response following one immunisation and was able to significantly prolong the survival of C57 BL/6 mice with established solid B16 tumours [58]. This study highlights the potential of ultrasound to improve the therapeutic response to TAAs in cancer models *in vivo*. To date, ultrasound has not been used to deliver TAAs in a preclinical prostate cancer model, however, several authors have employed ultrasound to enhance gene delivery to prostate cancer tumours *in vivo*.

Duvshani-Eshet *et al.* utilised ultrasound to enhance delivery of anti-angiogenic hemopexin-like domain fragment (PEX) genes to prostate tumours *in vivo* [59]. The group inoculated C57 BL/6 mice with PC-3 tumour cells and when the tumours reached 100 mm^3^ treatment was initiated with intratumoural (i.t.) injection of naked PEX expressing plasmid (pPEX) with or without therapeutic ultrasound (TUS). Tumour burden was monitored every 2 days for 28 days. Following a single application TUS significantly decreased tumour weight and volume compared to control (no treatment), 0.65 ± 0.15 g compared to 1.05 ± 0.25 g and 1300 ± 250 mm^3^ compared to 2000 ± 300 mm^3^, respectively. This effect was significantly improved by the addition of an ultrasound contrast agent, Optison. Optison is a microbubble composed of an albumin shell with a gas core used to enhance the ultrasound backscatter in the target tissue. Subsequent studies involved tumour implantation followed by weekly treatment for four weeks with pPEX and Optison with or without TUS. Repeated treatments of both pPEX or pPEX + Optison alongside TUS significantly reduced prostate tumour burden and growth by 80 % compared to a single treatment and control (no TUS). This study demonstrated the promise of ultrasound as a gene delivery strategy in cancer. However, while ultrasound can be easily directed towards specific tissues it is not always possible to inject therapy i.t. This limitation needs to be overcome before clinical translation of this delivery system can be achieved.

To conclude, ultrasound is a promising delivery option for the future, particularly for use in combination with other immunotherapeutic approaches. The non-invasive nature of ultrasound makes it an ideal candidate as a physical delivery system. At present the higher efficacy of other systems, such as EP, make them more appealing to researchers. EP and the gene gun have been used for DNA vaccination in numerous clinical trials and have well established safety profiles, however, ultrasound has not been evaluated to the same extent and the long term toxicity and efficacy still require elucidation.

#### Conclusions and future considerations

Physical delivery systems use the application of force to overcome the extra- and intra-cellular barriers to gene delivery. In the majority of systems this enables the bypassing of endocytosis and allows cellular entry of DNA through physically formed pores in the cell membrane. This leads to a rapid and sustained gene expression, thus, physical delivery systems represent a convenient and efficacious method for gene delivery *in vivo*. The advantages and disadvantages of these systems are summarised in Table 4. Delivery methods such as EP and the gene gun are well established in terms of efficacy, but are invasive and require the need for specialist equipment and training. This makes them less desirable for widespread vaccination use in a clinical setting. Ultrasound, while less invasive, suffers from a lack of efficacy compared to these established techniques and also requires specialist equipment. Further optimisation and investigation into the efficacy and cytotoxicity of this technique is required before it can be considered for routine use in gene delivery trials. However, ultrasound may have a role in enhancing the efficacy of gene delivery protocols when used in combination with other techniques. For example, Yamashita *et al.* [60] used a combination of EP and ultrasound, termed electro-sonoporation, to deliver plasmid DNA coding the luciferase reporter gene and mouse Interleukin-12 (mIL-12) to the quadriceps of mice. The group found that two days post delivery, mice that had undergone electro-sonoporation demonstrated luciferase expression levels two-fold higher than those that had received electroporation alone [60]. Likewise, levels of serum mIL-12 were found to be two-fold higher in mice treated with electro-sonoporation, with gene expression still detectable 28 days post administration [60].

**Table 4:** Summary of advantages and disadvantages of physical delivery strategies used in DNA vaccination

Physical delivery strategy	Advantages	Disadvantages
Electroporation	• High levels of transgene expression	• Invasive
	• Long-lasting gene expression	• Need for specialist equipment and training
	• Safety demonstrated in numerous clinical trials	• Potential for tissue damage
	• Commercially available delivery devices	• Two-step delivery process
Gene Gun	• High levels of transgene expression	• Invasive
	• Long-lasting gene expression	• Limited DNA carrying capacity
	• Safety demonstrated in clinical trials	• Need for specialist equipment and training
	• Commercially available delivery devices	• Often need for multiple administrations
	• One-step delivery process	• Low tissue penetration
Ultrasound	• Non-invasive	• Low levels of transgene expression
	• Can be targeted to specific organs easily	• Need for specialist equipment and training
		• Two-step delivery process
		• Safety not yet widely demonstrated in gene therapy clinical trials

This study highlights the potential in using a combination of delivery strategies to improve gene expression. While it is commonplace to enhance the immune response through the administration of biological or chemical adjuvants, new strategies are emerging using a combination of physical delivery systems to synergistically increase gene delivery [61–63]. These two-tier or combinational approaches are likely to yield a more efficacious gene delivery and thus, may prove necessary in larger animal models to produce sufficient amounts of antigen to overcome self-tolerance to TAAs.

#### DNA delivery vector

DNA delivery vectors can be broadly classified as viral or non-viral. DNA delivery vectors enhance the uptake of DNA and protect it from the intracellular barriers to gene delivery. This process involves condensing the DNA to facilitate endocytosis, masking the negative charge of the DNA and protecting it from degradation by nucleases. In addition, several delivery vectors are capable of directly trafficking DNA to the nucleus thus enhancing gene expression.

#### Viral vectors

Several types of viruses have been utilised as delivery vectors for DNA vaccines including adenoviruses (Ad) [64], adeno-associated viruses (AAVs) [65, 66], herpes simplex viruses (HSV) [67, 68], retroviruses [69], lentiviruses [70] and poxviruses [71]. Viruses have specifically evolved to overcome the barriers presented to gene delivery and as such, they are associated with high transfection efficacy and are the current gold standard for gene delivery [72]. A number of DNA vaccines using viral vectors have been used in prostate cancer preclinical and clinical trials and have proven safe and efficacious [67, 73–75].

Lubaroff *et al*. [74] recently reported encouraging results from a Phase I trial utilising an adenoviral vector to deliver DNA coding human PSA (Ad/PSA). Patients with evidence of metastatic castrate resistant disease received 1 × 10^6^, 1 × 10^7^ or 1 × 10^8^ CFU of Ad/PSA subcutaneously (s.c.) either as an aqueous suspension or as a Gelfoam collagen matrix [74]. Patients were then observed for adverse effects, and at days 14, 21 and 2, 4, 8 and 12 months returned for assessment and to allow evaluation of antibody or T cell specific responses to PSA. The group reported that 34 % of patients experienced an increase in detectable anti-PSA antibodies, while 68 % of patients developed anti-PSA T cells [74]. In addition, 46 % of patients experienced an increase in PSA-DT. The results of this small but encouraging study resulted in the commencement of a Phase II trial to assess the benefit of Ad/PSA in patients with recurrent prostate cancer [76], although results have yet to be published.

To date, perhaps the most successful prostate cancer DNA vaccination platform is that of the PSA-targeting vaccine, PROSTVAC, consisting of a prime-boost strategy with recombinant vaccinia virus and fowlpox virus vectors expressing PSA and a triad of co-stimulatory molecules, B7.1, ICAM-1 and LFA-3, known as TRICOM [71]. The success of a phase II clinical trial in men with metastatic CRPC demonstrated a survival benefit of 8.5 months in patients who received PROSTVAC-VF plus GM-CSF [75]. This has now led to a randomised double-blind phase III clinical trial [77].

Despite this success, a number of limitations have been highlighted with these vectors including time-consuming production, uncertain reproducibility, limited carrying capacity of transgenes, safety concerns such as toxicity, dose-dependent immunogenicity and potential integration into the host genome causing oncogene activation [36, 72]. Many groups continue to develop recombinant viral vectors due to their efficacy *in vivo*, and many DNA vaccines with these vectors continue to be brought to trial. However, there has been a shift towards creating new, non-viral vectors for DNA vaccination.

#### Non-viral vectors

Non-viral vectors, while attractive in terms of reproducibility and safety, are limited by low transfection efficacy *in vivo*. Existing vectors include cationic lipids, polymers and peptides [72]. Due to their cationic charge these vectors often spontaneously condense DNA to form smaller cationic nanoparticles in addition to enhancing endocytosis and protecting DNA from degradation. Despite ease of production, these vectors continue to suffer from a lack of efficacy compared to viral vectors *in vivo* and so the challenge is to improve non-viral characteristics to overcome the barriers to gene delivery.

#### Lipid/liposome delivery systems

Cationic lipids are capable of condensing DNA through electrostatic interactions into small lipoplexes. These lipoplexes carry a positive surface charge which aids internalisation through cell membrane binding. However, highly charged particles have been demonstrated to cause significant toxicity and aggregation with serum proteins which can hinder efficacy *in vivo*. Some of these limitations have been addressed through the functionalisation of liposomes by the addition of Poly-ethylene-glycol (PEG). PEG shields the liposome, increases the circulation time and facilitates the addition of ligands to improve targeting [78]. The addition of Mannose to liposomes has led to significant increases in transfection of DCs and macrophages through targeting of the mannose receptor [79–81]. Targeting of APCs makes these vectors ideal for DNA vaccination, and mannosylated liposomes have been demonstrated to enhance gene expression and the antigen specific immune response compared to non-mannosylated vectors [78, 79]. Liposome vectors have not been used for DNA vaccination purposes with prostate TAAs to date.

Allen *et al.* [82] delivered lipoplexes containing the gene coding for the p75 neurotrophin receptor (p75^NTR^) i.t. to PC-3 xenografts implanted on SCID mice. p75^NTR^ is a known tumour suppressor gene in prostate cancer and therefore it was hypothesised that transfection of established PC-3 tumours would result in an increase in cell apoptosis and a decrease in cell proliferation. The authors implanted SCID mice with 1x10^6^ PC-3 cells s.c. and 5 days post implantation injected i.t. with Lipofectamine/ p75^NTR^ cDNA (1 μg, 5 μg or 10 μg) or Lipofectamine 3 times weekly for 5 weeks [82]. Tumours treated with lipoplexes containing p75^NTR^ cDNA were significantly smaller than those treated with Lipofectamine or control (no treatment). In addition, tumour size was reduced in a dose dependent manner with 5 μg or 10 μg of DNA decreasing tumour volume significantly more than 1 μg of DNA [82]. The authors successfully demonstrated the feasibility of this approach for gene therapy for prostate cancer. However, while it was demonstrated that the lipoplexes were capable of transfecting prostate cancer cells in this model it is not always possible to administer i.t. *in vivo*, especially in diseases such as prostate cancer where there may be disseminated disease. Therefore, DNA vaccination protocols targeting prostate TAAs may be more relevant clinically, and do not require systemic delivery.

In conclusion, liposomes enable enhanced transfection through complexation with DNA, circumvent the tissue damage associated with physical delivery systems and do not require additional specialist equipment. Liposomes can be modified to enhance stability, improve circulation times and target APCs, making them good candidates for *in vivo* gene delivery. However, liposome vectors also continue to suffer from a lack of efficacy compared to viral vectors and cellular toxicity remains an on-going problem. Attempts to reduce the limitations of unspecific cellular transfection and poor circulation time through incorporation of PEG have also been undermined by the formation of PEG-specific antibodies upon repeat administration [72]. Liposome vectors still require further refinement before they become mainstream vectors for use in DNA vaccination.

#### Polymer delivery systems

Cationic polymers are also capable of condensing anionic DNA through electrostatic interaction to form particles known as polyplexes, and have been extensively studied as non-viral gene delivery agents. These synthetic polymers provide a simple method of gene delivery and are easily modified to accommodate other stabilising polymers, targeting ligands or drug conjugates [83]. Polyethylenimine (PEI) and poly (L-lysine) (PLL) have been the most widely studied cationic polymers. Although both PLL and PEI enhance DNA transfection, PEI is most effective. The large buffering capacity of PEI enables efficient endosomal escape via the ‘proton sponge effect’ [83]. However, the main limitation of these cationic polymers is that increased transfection efficacy is correlated to a higher molecular weight that results in a substantial increase in toxicity [83–85]. Attempts to reduce the toxicity of these polymers while maintaining the transfection efficacy are ongoing with varying success [86–88]. Polyplexes have not yet been used as non-viral vectors for DNA vaccine delivery in prostate cancer models, however several polyplexes have been used to deliver gene therapy to tumours in clinical and preclinical trials [89, 90]. Hence prostate cancer may benefit from gene therapy delivered in this manner.

Similar to liposomes, polymer carriers may be modified to contain mannose moieties that mediate delivery to APCs [61, 91]. Kim *et al.* [61] described a novel, two-tiered delivery system designed to enhance gene delivery to DCs in the dermal layer for DNA vaccination. The system utilised solid microneedles coated with a pH-responsive layer designed to release polyplexes when inserted into skin. Following insertion and release into the skin uptake to APCs may be accommodated by mannosylated polyplexes encoding an antigenic amyloid beta monomer, Aβ 1–42, which enhances APC uptake through interaction with mannose receptors. A single immunisation of BALB/c mice with 10 μg of DNA was sufficient to induce detectable Aβ-specific antibodies one-week post immunisation. Five weeks following immunisation mice challenged with Aβ 1–42 peptide produced a rapid and robust Aβ-specific humoral response, demonstrating the ability of this approach to induce a long-lasting antigen specific humoural response. This type of approach may prove beneficial in DNA vaccines for prostate cancer, where targeted transfection of APCs is key for the development of a robust cellular response to eradicate antigen expressing tumour cells.

In conclusion, while polymers provide a stable, efficacious vector for targeted gene delivery, further development is still required. Although unspecific cell targeting, poor circulation time and non-specific interaction of polyplexes with serum proteins have been largely improved through the incorporation of PEG and targeting ligands, cytotoxicity remains the rate limiting factor *in vivo*. Further development of newer polymers, not limited by a trade-off between efficacy and cytotoxicity is required before these vectors can be used widely in clinical trials for DNA vaccination.

#### Peptide delivery systems

In 1988 it was discovered that HIV TAT trans-activating factor was able to traverse the cell membrane and be taken up by a wide variety of cells [92]. This revelation led to the development of a whole class of natural and synthetic peptides capable of delivering cargo to a variety of cell types, known as cell penetrating peptides (CPPs) [93]. Furthermore, peptides are being developed that mimic viral sequences. These include peptides that facilitate internalisation [94], endosomal escape [95], and nuclear localisation [96]. Such peptides are often rich in basic amino acids such as lysine and arginine which are essential to condense DNA into nanoparticles via electrostatic interaction [95, 97]. The advantages of these viral mimetic peptides include biocompatibility, low cytotoxicity and versatility with respect to rational design resulting in tailored systems.

Zhang *et al.* recently demonstrated the feasibility of this approach in a mouse prostate cancer model where an in-house cationic peptide [K] 18P9, composed of 18 lysine residues and a human CTL PSCA epitope, was used to condense a plasmid encoding the full-length human PSCA (hPSCA) gene for immunisation. HLA-A2.1/Kb Tg mice were immunized with 25 nmol of DNA 3 times at 2 weekly intervals. Effector cells from the immunised mice were subsequently intravenously (i.v.) injected into tumour bearing nude mice once per week and tumour growth monitored. These results showed significant retardation in tumour growth in those receiving cells from peptide/DNA vaccinated animals compared to those immunised with DNA alone, thus confirming administration of the DNA vaccine complexed with this peptide elicited superior immune responses *in vivo* [98].

Peptide delivery systems confer a level of targeting and safety profile that is far superior to any other non-viral vehicle. Perhaps the drawback of peptide delivery systems lies in systemic administration where accumulation in the liver is a frequent event. However, given that most DNA vaccination strategies require either i.m. or i.d. injection, peptides are ideally placed to deliver their cargo to APCs and have the potential to fill that delivery void.

### Conclusions

Non-physical delivery methods carry the advantage of enhancing gene delivery in a non-invasive means to patients without requiring specialist equipment. Amongst the non-physical delivery systems viral vectors remain the gold standard in terms of efficacy. For DNA vaccination in larger animals and humans high transfection rates are essential, and as such, the extensive use and success of viral vectors for gene delivery in clinical trials makes them the most attractive vector for *in vivo* protocols. This success has led to the PSA-targeting DNA vaccine PROSTVAC entering Phase III clinical trials, the first prostate cancer specific DNA vaccine to do so. Despite these advantages there continues to be limitations over the safety, immunogenicity and carrying capacity of these vectors. Thus, there is a need to develop new, non-viral vectors capable of producing similar transfection efficacies. Unfortunately these vectors suffer from high toxicity and poor transfection rates *in vivo*, though modification with ligands to improve APC uptake is promising for DNA vaccination. Peptide delivery vectors hold promise in terms of being able to mimic viral characteristics for DNA delivery. However, there is still a significant gap in acquiring the necessary pre-clinical data to validate the peptide delivery of DNA TAAs.

### Co-stimulatory adjuvants

Co-administering immune enhancing molecules at the site of DNA vaccination either directly or encoded in plasmids is primarily designed to improve vaccine immunogenicity. Few studies directly compare the efficacy of DNA vaccines with and without adjuvant making it difficult to deduce the benefits of co-administration. In addition, there is little consensus as to whether these adjuvants should be delivered as soluble protein or as plasmids, making direct comparison between studies examining the effect of adjuvant problematic. The most commonly co-administered molecules include chemokines, cytokines and bacterial toxins [48].

GM-CSF is a cytokine commonly used as an adjuvant for DNA vaccination and has been used in numerous clinical trials [30, 31, 73, 99]. In a Phase I/II trial, Mincheff *et al.* demonstrated that 50 % of patients vaccinated i.d. at one weekly intervals with 100 μg of PSMA and CD86 encoding plasmid(s) showed signs of immunisation in the form of delayed-type hypersensitivity (DTH). In contrast, 100 % of patients vaccinated with PSMA and CD86 plasmids also receiving 40,000 IU of soluble GM-CSF i.d. showed signs of DTH upon challenge [100]. While this study demonstrated that GM-CSF was capable of enhancing the general immune response to DNA vaccination, it is unclear as to whether GM-CSF enhanced the antigen specific response or improved patient outcomes. As such, more studies directly comparing the effect of GM-CSF on the antigen-specific cellular and humoral immune responses induced by prostate cancer DNA vaccines are necessary. Indeed, current studies with the PROSTVAC vaccine include a phase III randomized, double-blind trial to examine the effect with and without GM-CSF [77].

Several preclinical studies have demonstrated the benefits of the co-expression of GM-CSF with DNA vaccines for tuberculosis [101], encephalitis [102], and melanoma [103]. However, these studies highlighted that co-inoculation with plasmid GM-CSF did not confer the same benefits, possibly due to unpredictable GM-CSF expression and competition with plasmid DNA encoding antigens for cellular uptake. Therefore, when considering the benefit of GM-CSF as an adjuvant it is crucial to consider the mode of GM-CSF delivery and demonstrate a clear benefit of inclusion.

### Xenogeneic DNA

A number of TAAs possess functional homologues in other animal species, where the expression patterns and functions are similar to those of their human counterparts [104, 105]. As well as providing suitable preclinical models for DNA vaccines in a “self” model of prostate cancer, these xenoantigens have been used by groups in an effort to increase the immunogenicity of DNA vaccines. As xenoantigens are highly homologous to native peptides, they can be capable of eliciting a specific cross-reactive response towards the host self-antigen that can overcome tolerance issues.

Johnson *et al.* immunised Lewis rats with naked pTVG-HP, a DNA plasmid encoding full length human PAP (hPAP), at 2 weekly intervals, for up to six i.d. immunisations with doses of 100 μg, 500 μg or 1500 μg of pTVG-HP [37]. Immunisation with pTVG-HP elicited hPAP-specific CD4+ and CD8+ T cells at the lowest dose following two immunisations. PAP-specific IgG antibodies were also detectable in all pTVG-HP treated animals and levels increased with increasing DNA dose and frequency of immunisations [37]. Further work from this group immunising rats with pTVG-RP, encoding rat PAP (rPAP) elicited hPAP-specific T cell responses following 6 immunisations indicating a larger number of immunisations are necessary to elicit a cross-reactive immune response in this model [106].

To confirm the animals immunised with pTVG-RP elicited a rPAP specific response in addition to hPAP specific responses splenocytes were isolated from the animals and restimulated *in vitro* with hPAP or rPAP expressing DCs and the resultant levels of interferon gamma (IFN-ϒ) were analysed. Immunisation with pTGV-RP did elicit a rPAP-specific T cell response and a cross-reactive hPAP immune response. This indicates that autologous PAP antigen is capable of overcoming tolerance to autologous PAP [106]. Based on these preclinical data, and using the same immunisation schedule, the group carried out a Phase I/IIa clinical trial in 22 patients with D0 prostate cancer [31]. Patients were immunised with pTVG-HP, a self-antigen in humans. Responses were found in 9 of the 22 patients with a tripling in PAP-specific CD4+ or CD8+ T cell responses, and 7 of the 22 patients experienced at least doubling of the PSA-DT [31]. Upon completion of the initial study, two patients who had developed CD8+ T-cell responses to PAP received monthly booster vaccinations of 100 μg pTVG-HP to determine whether this could augment the initial immune response [107]. Prior to continuation of treatment neither patient had residual, detectable PAP-specific T cells, and upon only two booster immunisations one patient developed detectable levels of CD4+ and CD8+ PAP-specific T cells suggesting that further immunisations could indeed prove beneficial [107].

The group hypothesised that immunisation with a xenoantigen may require lower numbers of immunisations to elicit immune responses against the host antigen, and carried out immunisation of Lewis rats with pTVG-HP to determine whether rPAP could be targeted through a cross-reactivity to immunisation with the human antigen [108]. The group found that despite the high homology between rat and human PAP peptides the xenoantigen was not capable of eliciting a cross-reactive immune response to native rPAP [108], suggesting that vaccination of humans with xenoantigens may not produce an advantageous immune response against the native antigen. The authors suggested that this may not be the case with all xenoantigens, as the major determinant of whether a foreign peptide is capable of inducing a cross-reactive immune response may not be the overall homology of the foreign and self peptides, but the homology of the epitopes presented to the immune system [108].

Following the promising results of their Phase I/IIa clinical trial and to evaluate the benefit of further “booster” immunisations, 17 patients were recruited into a Phase II clinical trial to assess the safety, immunological impact and clinical outcome of pTVG-HP, administered with rhesus macaque GM-CSF (rhGM-CSF) as an adjuvant, in patients with non-metastatic castrate resistant disease [99]. Patients were randomised to receive 100 μg pTVG-HP with 200 μg rhGM-CSF i.d. biweekly for 12 weeks and then subsequent boosters every 12 weeks until radiographic progression (Group 1) or boosters every 2, 4 or 12 weeks depending on cellular immune response (Group 2). From Group 1, 3 of 8 patients experienced at least a tripling in PAP-specific T cells compared to 6 of 8 participants from Group 2 [99]. Additionally, 3 of 8 patients from Group 1 and 4 of 9 patients from Group 2 experienced at least a doubling in their PSA-DT [99]. Taken together these results suggest that further periodic booster immunisations are of benefit to develop an immunological response and do not result in tolerance to the targeted antigen.

Several other groups have reported encouraging results using xenoantigens, Castelo-Blano *et al.* treated mice bearing TRAMP-C2 prostate tumours over 10 days with 4 intraneoplastic injections of 1x10^7^ PFU of oncolytic herpes simplex virus (oHSV) constructs expressing human PAP or mouse PAP (mPAP). Mice immunised with oHSV expressing hPAP had significantly reduced tumour growth and prolonged survival compared to those treated with oHSV expressing mPAP (*p* = 0.01) or control (*p* = 0.0008), with a 10 day prolongation of survival to 39 days compared to the latter [67]. Furthermore Gregor *et al.* demonstrated that 5 intraperitoneal (i.p.) immunisations of mice with human PSMA (hPSMA) encoding DNA vaccine or protein was capable of inducing autoantibodies against native mouse PSMA (mPSMA) as demonstrated by ELISA. However, immunisation with native PSMA encoding DNA vaccine elicited no detectable increase in immune response to human or murine PSMA [55]. Moving from this preclinical study Slovin *et al.* carried out a Phase I trial immunising patients with metastatic prostate cancer at 3 week intervals i.m. with 100 μg, 1500 μg or 4000 μg of xenogeneic or homologous DNA vaccine coding PSMA [109]. Patients received three immunisations and subsequently patients who had received homologous vaccination were immunised a further three times with xenogeneic DNA The vaccine has proven safe, however, no high titer antibodies specific to PSMA were produced by any patients, though further analysis of T cell reactivity is ongoing but has yet to be published. As such, further studies are required to elucidate whether xenogeneic or autologous DNA is optimal in the clinical setting.

### Prime / boost strategies

Many DNA vaccines have proven efficient at elicitation of immune responses, however, in some cases these responses are suboptimal to provide protection against the antigen. Thus, a number of immunisation regimens involving “priming” with DNA and subsequently “boosting” with a heterologous agent such as a different antigen delivery platform or protein with the aim of improving immunogenicity have been investigated.

This approach has recently been explored using murine STEAP1 (mSTEAP1) and murine PSCA (mPSCA) in mice for prophylaxis of prostate cancer [17]. The group primed mice with recombinant DNA (mSTEAP1 and/or mPSCA) and boosted with modified vaccinia virus ankara (MVA) vector expressing the same antigen(s). The group found that in a tumour challenge study, mice immunised against either antigen using this protocol demonstrated significantly inhibited tumour growth compared to control, 49.8 % following immunisation against mPSCA and 41.7 % against mSTEAP1 [17]. Another group immunised mice against mSTEAP using several vaccination protocols including a gene gun mSTEAP prime/ s.c. mSTEAP-Virus Replicon Particle (VRP) boost; gene gun mSTEAP prime/ s.c. mSTEAP DNA boost and mSTEAP-VRP prime and boost [56]. Mice were then challenged 10 days post boost with TRAMP C-2 prostate cancer cells. Tumour growth was monitored twice weekly and survival followed until tumours reached volumes over 1000 mm^3^. While survival was significantly increased with all mSTEAP vaccination protocols, the most significant effect was seen in mice vaccinated with mSTEAP DNA and boosted with mSTEAP-VRP. In a phase I/II trial Mincheff *et al.* found that all patients immunised with a replication deficient adenoviral vector expressing PSMA and later boosted with plasmid PSMA showed signs of immunisation (by DTH), while only 50 % of patients vaccinated with plasmid PSMA/CD86 alone showed signs of immunisation [100]. Indeed PROSTVAC employs two different viral vectors, upon priming with the recombinant vaccinia virus expressing PSA, neutralizing antibodies are formed to the vector, making subsequent boosting doses unfeasible. However, boosting with a fowlpox vector overcomes this limitation [71] and similar strategies should be implemented in the design of new DNA vaccines.

Priming with DNA and boosting with protein has also proven successful. Gregor *et al.* immunised C57 BL/6 mice with a DNA vaccine encoding hPSMA once weekly for 5 weeks. Sera from these animals was subsequently analysed for affinity to mPSMA. The mouse with the best response was subsequently boosted with 10 μg hPSMA protein and found resultant antibody specificity for hPSMA and cross-reactivity for mPSMA [55]. A preclinical study carried out by Yong *et al.* demonstrated that C57 BL/6 mice immunised with a DNA vaccine encoding gastrin-releasing peptide (GRP) (three doses of 50 μg DNA), followed by boosting with HSP65-GRP6 protein resulted in increased immunogenicity as compared to those receiving DNA vaccination alone, as indicated by an increased titre of anti-GRP antibodies and inhibition of prostate tumour growth (tumour weight 0.962 ± 0.462 and 1.536 ± 0.497 g respectively) [110].

### Multivalent strategies

Several recent preclinical studies in rodents have focused on the effects of DNA vaccines coding multiple TAAs, the authors hypothesise that delivering multiple TAAs will result in a wider and more potent immune response, targeting tumour cells with a synergistic effect [12]. Ferraro *et al.* used a dual antigen approach to immunise mice, PSA and PSMA were co-delivered i.m. followed by EP. The immunisation elicited a robust vaccine-specific CD4+ and CD8+ T cell response, indicating that the approach may hold clinical promise [12]. In the tumour challenge study carried out by Krupa *et al.* described previously [17], the DNA prime/MVA boost strategy significantly delayed tumour growth upon challenge with TRAMP C-1 tumours. At day 55, the most significant effect was seen in mice immunised with both mSTEAP1 and mPSCA, with tumour volume 76.5 % lower than control mice vaccinated with empty plasmid vectors. The group chose to immunise TRAMP mice (a more relevant and aggressive prostate cancer model) using this prime/boost strategy. The vaccinated mice had significantly reduced primary tumour burden and at 24 weeks showed lower histological grade tumours, indicating that this approach is capable of breaking tolerance to self-antigens [17]. While these preclinical data demonstrate promise for this strategy an earlier preclinical study by Kim *et al.* primed mice with recombinant adenoviruses expressing mSTEAP, mPSCA and mPSMA and then boosted with DCs pulsed with tumour lysate [111]. This prime/boost strategy effectively delayed tumour growth following TRAMP C-1 tumour challenge, however, only high levels of mSTEAP-specific CD8+ T cells were found in the blood and spleens indicating that the anti-tumour effect was predominantly due to mSTEAP vaccination. Furthermore, it was found that vaccinating against mSTEAP alone was as effective as the triple antigen approach [112]. These results suggest that the use of multiple TAAs, while demonstrating promise in some studies is not generalisable, and *in vitro* and preclinical models should be used to determine the most effective combination of TAAs for each individual delivery platform.

## Conclusions

As discussed, DNA vaccination represents a promising platform capable of offering both prophylactic protection and therapeutic treatment of prostate cancer. Currently there are a significant number of pre-clinical and clinical trials underway utilising a range of DNA plasmids, encoding a variety of TAAs and being delivered by an array of delivery methods. However, there are a number of questions still to be answered in order to achieve optimal immune responses clinically following vaccination. Primarily, is there an optimal dose of DNA and dosing regimen capable of provoking strong cellular and humoural responses to the antigens produced while avoiding exacerbation of pro-inflammatory responses to the vaccine and delivery vehicles utilised themselves.

Additionally, improved formulation and superior delivery technologies are essential for the enhancement of clinical data which has thus far proved disappointing when compared to the promising results elicited *in vitro* and in rodent models *in vivo*. It is becoming increasingly common for researchers to use a combination of delivery strategies to achieve higher transfection rates *in vivo*. These two-tier delivery systems enable synergy between the delivery systems and may prove necessary to achieve sufficient gene expression to provoke robust cellular and humoral immune responses [58, 61–63]. Among these strategies the use of microneedles to localise DNA delivery into the APC rich dermal layer has been commonly reported [61–63]. These devices provide a non-invasive means of overcoming the *Stratum Corneum* and do not require specialist training for use. This provides a significant benefit over other delivery platforms, especially where repeated administration or large scale vaccination is required. In conclusion, further investigation and advancement in the use of DNA vaccination for the protection against, and treatment of prostate cancer is necessary before a fully validated prostate cancer vaccine is clinically available.

## Abbreviations

AAV: Adeno-associated viruses
Ad: Adenovirus
APC: Antigen-presenting cell
CRPC: Castration resistant prostate cancer
CTLs: Cytotoxic T lymphocytes
DNA: Deoxyribonucleic acid
DOM: Domain of fragment C tetanus toxin
DTH: Delayed-type hypersensitivity
EP: Electroporation
FDA: US food and drug administration
GM-CSF: Granulocyte macrophage- colony stimulating factor
GRP: Gastrin-releasing peptide
hPSA: Human prostate specific antigen
hPSMA: Human prostate specific membrane antigen
HSV: Herpes simplex virus
I.D.: Intradermal
IFN-γ: Interferon gamma
I.M.: Intramuscular
I.P.: Intraperitoneal
I.T.: Intratumoural
hPAP: Human prostatic acid phosphatase
MHC: Major histocompatibility complex
mPSCA: Murine prostate stem cell antigen
mPSMA: Murine prostate specific membrane antigen
mSTEAP: Murine six transmembrane epithelial antigen of the prostate
MVA: Modified Vaccinia virus ankara
oHSV: Oncolytic herpes simplex virus
p75^NTR^: p75 neurotrophin receptor
PAP: Prostatic acid phosphatase
PEG: Poly-ethylene-glycol
PEX: Antiangiogenic hemopexin-like domain fragment
PSA: Prostate specific antigen
PSA-DT: Prostate specific antigen doubling time
PSCA: Prostate stem cell antigen
PSMA: Prostate specific membrane antigen
rhGM-CSF: Rhesus macaque granulocyte macrophage- colony stimulating factor
rPAP: Rat prostatic acid phosphatase
rhPSA: Rhesus macaque prostate specific acid
S.C.: Subcutaneous
STEAP: Six transmembrane epithelial antigen of the prostate
STEAP1: Six transmembrane epithelial antigen of the prostate 1
TAAs: Tumour associated anitgens
TRAMP: Transgenic adenocarcinoma of the mouse prostate
TUS: Therapeutic ultrasound
VRP: Virus replicon particle
